# Tumor Necrosis Factor Inhibition and Head and Neck Cancer Recurrence and Death in Rheumatoid Arthritis

**DOI:** 10.1371/journal.pone.0143286

**Published:** 2015-11-23

**Authors:** Christopher Phillips, Angelique L. Zeringue, Jay R. McDonald, Seth A. Eisen, Prabha Ranganathan

**Affiliations:** 1 Washington University School of Medicine, St. Louis, Missouri, United States of America; 2 St. Louis Veterans Affairs Medical Center, St. Louis, Missouri, United States of America; University of Kentucky, UNITED STATES

## Abstract

The objective of this retrospective cohort study was to determine the effect of tumor necrosis factor inhibitor (TNFi) therapy on the risk of head and neck cancer (HNC) recurrence or HNC-attributable death in patients with rheumatoid arthritis (RA). RA patients with HNC were assembled from the US national Veterans’ Affairs (VA) administrative databases, and diagnoses confirmed and data collected by electronic medical record review. The cohort was divided into those treated with non-biologic disease-modifying anti-rheumatic drugs (nbDMARDs) versus TNF inhibitors (TNFi) after a diagnosis of HNC. Likelihood of a composite endpoint of recurrence or HNC-attributable death was determined by Cox proportional hazards regression. Of 180 patients with RA and HNC, 31 were treated with TNFi and 149 with nbDMARDs after the diagnosis of HNC. Recurrence or HNC-attributable death occurred in 5/31 (16.1%) patients in the TNFi group and 44/149 (29.5%) patients in the nbDMARD group (p = 0.17); it occurred in 2/16 (13%) patients who received TNFi in the year prior to HNC diagnosis but not after. Overall stage at diagnosis (p = 0.03) and stage 4 HNC (HR 2.49 [CI 1.06–5.89]; p = 0.04) were risk factors for recurrence or HNC-attributable death; treatment with radiation or surgery was associated with a lower risk (HR 0.35 [CI 0.17–0.74]; p = 0.01 and HR 0.39 [CI 0.20–0.76]; p = 0.01 respectively). Treatment with TNFi was not a risk factor for recurrence or HNC-attributable death (HR 0.75; CI 0.31–1.85; p = 0.54). We conclude that treatment with TNFi may be safe in patients with RA and HNC, especially as the time interval between HNC treatment and non-recurrence increases. In this study, TNF inhibition was not associated with an increase in recurrence or HNC-attributable death.

## Introduction

Head and neck cancer (HNC) is a relatively common entity in the veteran population. Its frequency likely reflects the high prevalence of tobacco and alcohol use in this group, two well-known risk factors for this type of malignancy [1]. Treatment with tumor necrosis factor inhibitors (TNFi) in patients with rheumatoid arthritis (RA) increases the risk of certain cancers. We and others have reported on the increased risk of non-melanoma skin cancer in patients with RA treated with TNFi compared to those treated with non-biologic disease-modifying anti-rheumatic drugs (nbDMARDs) [2–5]. However, the effect of TNFi on the natural history of individual solid tumors such as HNC has not been adequately examined.

Rheumatologists are often faced with difficult clinical situations regarding the potential risks and effects of immunosuppression on an individual patient’s comorbidities including a history of malignancy. In the case of HNC, which is strongly associated with human papilloma virus infection, there is reason for additional concern as immunosuppression may potentially play a role in accelerating the natural history of the cancer. Hence a systematic analysis of the impact of TNF antagonism on the natural history of HNC will help guide rheumatologists in the management of patients with RA and a history of HNC.

The United States (US) national Veterans’ Affairs (VA) administrative databases offered the opportunity to assemble a large cohort of patients with both RA and HNC, to examine this issue. We hypothesized that TNFi used in patients with a known diagnosis of HNC may increase the risk of recurrence or HNC-attributable death. Among patients with RA who had been diagnosed with HNC, we examined the risk factors for a composite endpoint of recurrence or HNC-attributable death, with a particular interest in the effect of TNFi therapy on this outcome. The goal of our study was to determine the impact of TNF antagonism on HNC recurrence or HNC-attributable death in patients with RA.

## Methods

### Data Sources

This study was approved by the institutional review board of the St. Louis VA medical center. We acquired data from the VA’s Austin Information Technology Center (AITC) and the Pharmacy Benefits Management (PBM) databases, which contain the VA’s centralized national administrative data. AITC data included all inpatient and outpatient International Classification of Diseases, Version 9, Clinical Modification (ICD-9-CM) diagnosis codes, encounter data, and demographic data. PBM data included all inpatient and outpatient pharmacy data. Data from both the AITC and PBM were merged into a single database. Patients identified with possible RA and HNC from this database subsequently underwent review of electronic medical records using the Compensation and Pension Records Interchange (CAPRI), an electronic system that can be used to access individual patient electronic medical records at a national level in the VA healthcare system. CAPRI review was performed to confirm the diagnoses of RA and HNC, and to collect additional variables not available from the national VA administrative databases. All patient information was anonymized and de-identified prior to analysis.

### Study Cohort

We constructed our cohort of veterans with RA and HNC in two steps. In the first step, we screened VA national administrative databases for veterans who met the following criteria between October 1, 1998 and September 30, 2008: 1) received an ICD-9-CM diagnosis code of RA, 2) received at least one prescription for a DMARD from the VA, 3) had at least a 4-month history of receiving medication from the VA prior to first DMARD prescription (in order to identify the date of first RA treatment) 4) had at least two separate clinic visits during the study period (to allow for follow-up), and 5) had an ICD-9-CM code for HNC. In the second step, the electronic medical records of all veterans meeting these criteria were reviewed by the first author (CP), a board-certified internist and rheumatology fellow, to verify the presence of both RA and HNC. Subjects who were not confirmed by electronic medical record review to have both RA and HNC during the study period were excluded. The final cohort consisted of all patients with a confirmed diagnosis of both RA and HNC during the study period, who received a DMARD after diagnosis of HNC.

### Definitions

#### Rheumatoid Arthritis (RA)

We screened for RA using an algorithm validated by Singh et al [6], requiring both of the following from administrative data: 1) the occurrence of an ICD-9-CM code for RA (714.0, 714.1, 714.2, or 714.81) on at least one occasion in either the inpatient or outpatient record, and 2) the receipt of a prescription for a DMARD on at least one occasion. Electronic medical records of patients identified using this algorithm were reviewed, and RA diagnosis was confirmed if a physician noted that a patient had RA in the narrative text or in a written problem list. RA was considered present if the patient was noted to have “likely” or “probable” RA, but not if the qualifier was “possible,” “suggestive of” or if the diagnosis was presented in a differential diagnosis format (i.e., RA vs. osteoarthritis), or if the diagnosis of RA was later found to be erroneous.

#### Disease Modifying Anti-Rheumatic Drugs (DMARDs)

DMARDs were characterized as nbDMARDs and TNFi. The following drugs were defined as nbDMARDs: hydroxychloroquine, methotrexate, sulfasalazine, leflunomide, azathioprine, cyclophosphamide, cyclosporine, oral and injectable gold, and penicillamine. TNFi included etanercept, infliximab, and adalimumab. A patient ever treated with a TNFi after HNC diagnosis was considered TNFi-exposed.

#### Head and Neck Cancer (HNC)

We screened for HNC by looking for a single occurrence of the following ICD-9-CM diagnosis codes in VA administrative data: ICD-9-CM codes for cancer of lip, tongue, major/minor salivary glands, gum, mouth, oropharynx, nasopharynx, hypopharynx (140.0–149.9, 195.0, 196.0), carcinoma in situ (230.0, 231.0), cancer of the nasal cavity, middle ear, sinuses, larynx (160.0–161.9). Electronic medical records of patients identified using this algorithm were subsequently reviewed, and HNC was considered present if a clinician stated in a narrative note that the patient had ever had HNC, or if a biopsy showed HNC (including carcinoma of the lip, tongue, major/minor salivary glands, gum, mouth, oropharynx, nasopharynx, hypopharynx, nasal cavity, middle ear, sinuses, or larynx). HNC histology was determined based on pathology or the narrative text of clinician notes. Narrative text of clinician notes describing HNC as the “probable” or “likely” diagnosis were accepted as a diagnosis of HNC; narrative text of clinician notes describing HNC as “possible” was not accepted as a diagnosis of HNC.

#### Covariates

Covariates obtained through complete review of VA electronic medical records included age, race, gender, smoking (ever/never at the time of HNC diagnosis) and alcohol use (current/not current at the time of diagnosis), indicators of RA severity (extra-articular disease, history of joint replacement, and rheumatoid factor positivity), and stage and treatment modality of HNC (chemotherapy, radiation, surgery). Covariates obtained through VA administrative databases included elements of the modified Romano implementation of the Charlson comorbidity index [7], and history of prior malignancy.

#### Outcomes

Recurrence was defined as progressive HNC after prior treatment. Recurrence was determined by medical record review. A patient was considered to have recurred if an oncologist used the term “recurrence” or “progression,” or if subsequent staging showed an advance in stage. Death was considered to be HNC-attributable if the death would most likely not have occurred in the absence of HNC. A patient was considered to meet our primary composite outcome of HNC recurrence or HNC-attributable death if the patient had HNC recurrence, HNC-attributable death, or both.

### Statistical Analysis

Multiple Cox regression was used to perform time-to-event analyses including a multivariate model examining risk factors for recurrent HNC or HNC-attributable death using time-varying covariates. TNFi therapy status was modeled as a time-varying covariate, though only three subjects switched into the TNFi exposure group post- HNC diagnoses. All other risk factors including smoking and alcohol use were not modeled as time-varying covariates due to the sporadic and inconsistent documentation of smoking status and alcohol use in the medical records. Multivariate survival regression models using time-varying covariates do not assume proportional hazards, hence this assumption was not tested. Chi-square, t-tests, and Wilcoxon rank sum tests were used to assess baseline differences between the groups. The three subjects who switched treatment groups were included in the TNFi group for these comparisons. A two-sided p-value of <0.05 was considered statistically significant. Outcome risk was described using hazard ratios (HRs). All analyses were performed using SAS software version 6.12 (SAS Institute, Cary, NC). Kaplan-Meier time-to-event curves were created using R software version 2.5.1 (R Foundation, Vienna, Austria).

## Results

**Fig 1:**
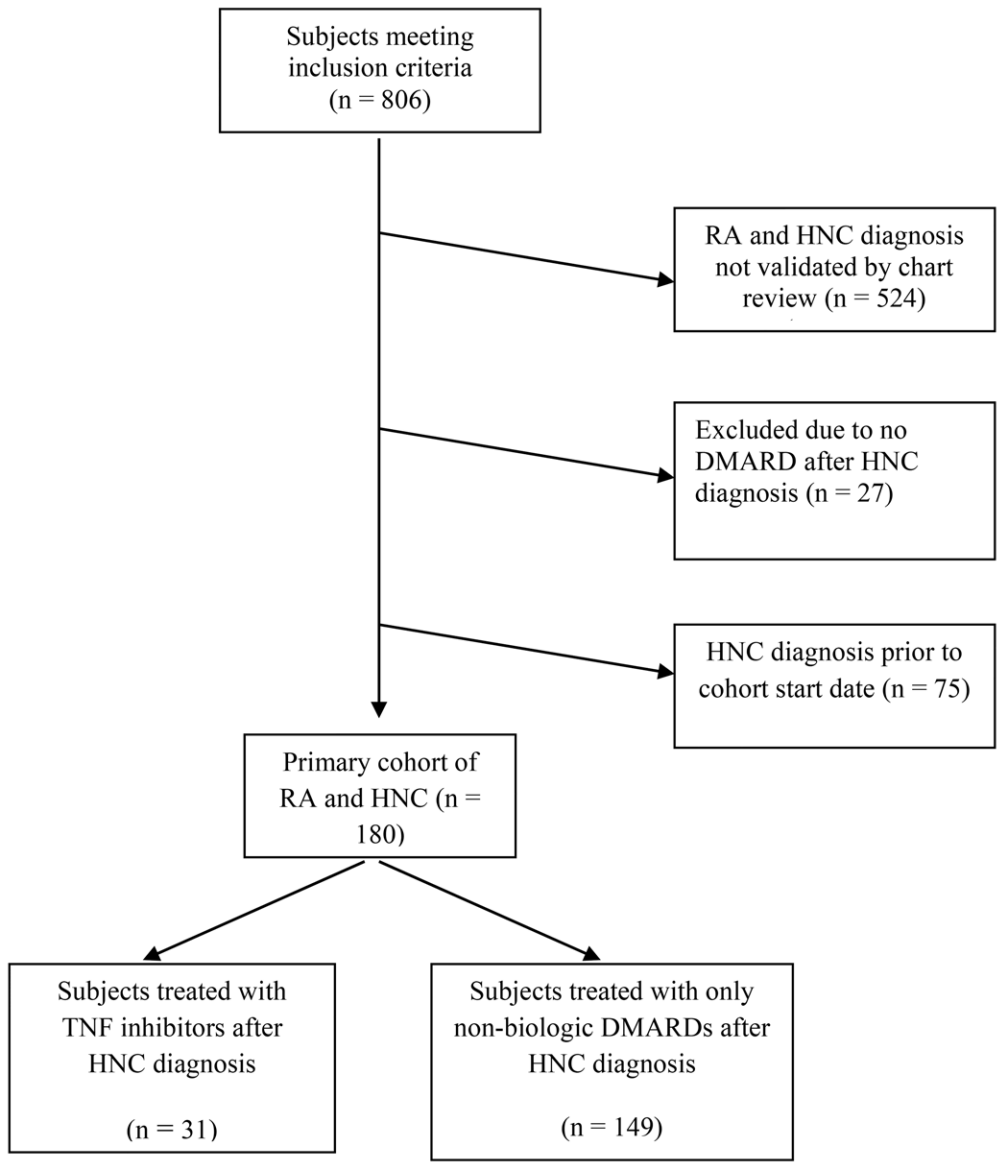
Study flow diagram.

Of the 806 patients identified through the VA administrative databases with ICD-9-CM codes for RA and HNC, 524 were excluded because the diagnoses of RA and HNC were not confirmed by electronic medical record review. Twenty-seven were excluded because they received no DMARD after HNC diagnosis. None were excluded because age, gender, or race could not be determined. Two hundred and fifty-five patients had both RA and HNC after validation by electronic medical record review. Of these, 75 patients had HNC diagnosis prior to the cohort start date of October 1, 1998 and were excluded. Our final cohort included 180 patients with both RA and HNC. Of these, 31 patients were treated with TNFi after the diagnosis of HNC, and 149 were treated only with nbDMARDs after HNC diagnosis (Fig 1). Of the 31 who received TNFi after HNC diagnosis, 28 had received TNFi before the diagnosis of HNC as well. Of note, 16 subjects in the nbDMARD group were treated with TNFi within one year prior to HNC diagnosis and not subsequently treated with these agents, likely reflecting rheumatologists’ concern that treatment may accelerate the natural history of HNC.

Demographic and clinical characteristics of these patients are presented in Table 1. The cohort was predominantly male, Caucasian and over 60 years of age with a high prevalence of smoking and alcohol use. The Romano score for co-morbidities, although slightly higher in the nbDMARD group (mean 5.4) compared to the TNFi group (mean 3.9), was not significantly different (p = 0.07) between the groups. Markers of RA disease severity such as the presence of extra-articular RA (p = 0.69), history of joint replacement (p = 0.75), and rheumatoid factor positivity (p = 0.52) were similar between the groups. Current alcohol use was the only socio-demographic variable which was significantly different between the two groups (67.7% TNFi vs. 43.6% nbDMARD, p = 0.04). Patients were diagnosed with HNC at a mean of 12.6 years after their diagnosis of RA in the nbDMARD group compared to 12.3 years in the TNFi group; this difference reached borderline significance (p = 0.05).

**Table 1:** Demographic and clinical characteristics of RA patients with head and neck cancer.

	Treatment after diagnosis of head and neck cancer		
Characteristics	TNF inhibitor therapy*	Non-biologic DMARDs **	p-value
N	31	149	
Mean age in years (SD)	63.9 (7.8)	66.0 (9.1)	0.24
Sex (% male)	31 (100%)	147 (98.7%)	0.52
Race (% Caucasian)	28 (90.3%)	122 (81.9%)	0.38
Current smoking (%)	16 (51.6%)	85 (58.6%)	0.61
Ever smoking (%)	28 (90.3%)	141 (94.6%)	0.50
**Current alcohol (%)**	**21 (67.7%)**	**65 (43.6%)**	**0.04**
Ever alcohol (%)	27 (87.1%)	111 (74.5%)	0.30
Mean modified Romano score (SD)	3.9 (4.5)	5.4 (4.1)	0.07
Extra-articular RA (%)	5 (16.1%)	17 (11.4%)	0.69
Joint replacement (%)	5 (16.1%)	18 (12.1%)	0.75
Rheumatoid factor positive (%)	23 (74.2%)	98 (65.8%)	0.52
Mean years from RA diagnosis to head and neck cancer diagnosis (SD)	12.3 (10.8)	12.6 (12.4)	0.05‡
Prior malignancy (%)	14 (45.2%)	88 (59.1%)	0.16
Chemotherapy (%)	10 (32.3%)	53 (35.6%)	0.73
Radiation (%)	25 (80.7%)	111 (74.5%)	0.47
Surgery (%)	16 (51.6%)	77 (51.7%)	0.99
Remission (%)	27 (87.1%)	110 (73.8%)	0.11
Mean months from head and neck cancer diagnosis to recurrence or HNC-attributable death	17.0 (7.4)	16.7 (13.1)	0.59‡
Head and neck cancer recurrence or HNC-attributable death (%)	5 (16.1%)	44 (29.5%)	0.17

* Exposure to TNFi therapy after head and neck cancer diagnosis assigns subjects to this group irrespective of whether they were exposed to TNFi therapy prior to the malignancy diagnosis

** Exposed only to non-biologic DMARDs after head and neck cancer diagnosis

‡Wilcoxon Rank-sum Test

There were no differences in HNC stage at the time of first diagnosis between the two groups (p = 0.64). Stage 1 was the presenting stage for 19.4% of those in the TNFi group and 22.1% of those in the nbDMARD group; percentages were similar for stage 2 (25.8% vs 15.4%), stage 3 (6.4% vs 12.1%), stage 4 (32.2% vs 34.9%), and unknown (16.1% vs 15.4%). Among the 16 subjects treated with TNFi prior to but not after HNC diagnosis, there were 3 subjects in stage 1 (19%), 3 in stage 2 (19%), 6 in stage 3 (38%), 3 in stage 4 (19%), and 1 unknown stage (6%) (percentages rounded). There were no differences between the two groups in the treatment modalities for HNC including chemotherapy (p = 0.73), radiation (p = 0.47), and surgery (p = 0.99).

HNC recurrence or HNC-attributable death occurred at a mean of 17.0 months after initial diagnosis of HNC in the TNFi group, and 16.7 months after initial diagnosis in the nbDMARD group (p = 0.59). HNC recurrence or HNC-attributable death occurred in 5/31 (16.1%) in the TNFi group and 44/149 (29.5%) in the nbDMARD group (p = 0.17). Among the three subjects who received TNFi after but not before HNC diagnosis, one subject had recurrence and HNC-attributable death. Among the 16 subjects who received TNFi before but not after HNC diagnosis, there were 2 subjects who had recurrence and HNC-attributable death. Details of the duration of follow-up of HNC in terms of time to recurrence or death andcensorship in the two groups are shown in Table 2.

**Table 2:** Duration of follow-up of head and neck cancer in the two treatment groups.

Time to recurrence or HNC-attributable death in months	Mean (SD)	Median	Minimum	Maximum
TNF inhibitor therapy	13.5 (7.9)	12	2	26
Non-biologic DMARD therapy	11.3 (10.6)	9	0.4	66
**Time to censorship in months**				
TNF inhibitor therapy	40.3 (28.2)	38	1	93
Non-biologic DMARD therapy	42.4 (32.8)	31	1	127

**Fig 2:**
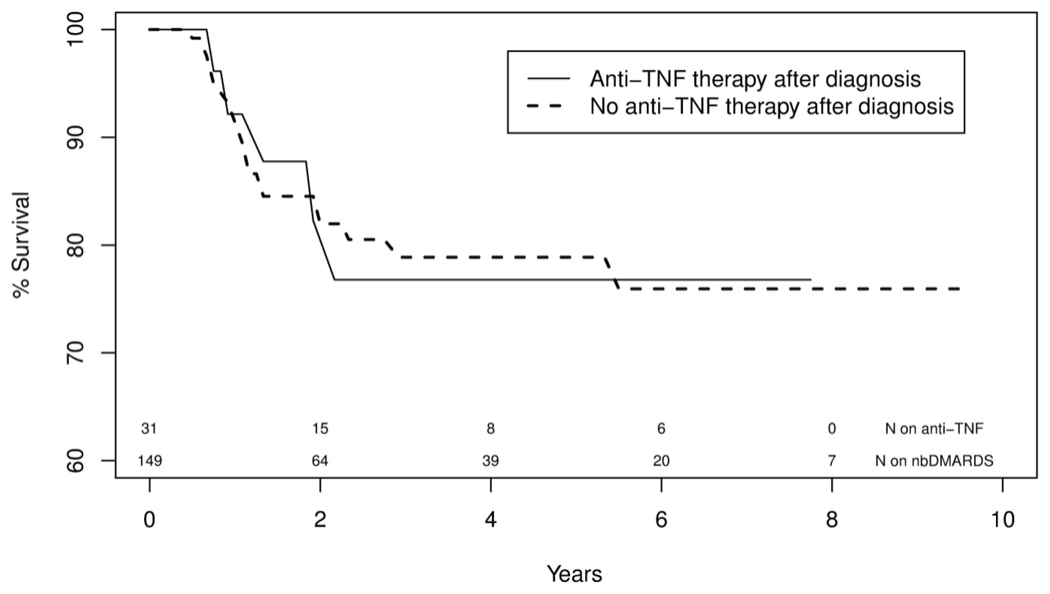
Survival curves for a composite outcome of head and neck cancer (HNC) recurrence or HNC-attributable death in RA patients exposed to TNF inhibitor therapy and non-biologic (nb) DMARDs.

In an adjusted, multivariate model, overall stage at diagnosis (p = 0.03) and stage 4 cancer were risk factors for recurrence or HNC-attributable death (HR 2.49 [CI 1.06–5.89]; p = 0.04); treatment with radiation or surgery was associated with a lower risk of recurrence or HNC-attributable death (HR 0.35 [CI 0.17–0.74]; p = 0.01 and HR 0.39 [CI 0.20–0.76]; p = 0.01 respectively) (Table 3). Exposure to TNFi was not a risk factor for our primary composite outcome of HNC recurrence or HNC-related death (HR 0.75; CI 0.31–1.85; p = 0.54) (Table 3 and Fig 2).

**Table 3:** Risk factors for head and neck cancer recurrence or HNC-attributable death in multivariate analysis.

Risk factors		Recurrence/ death	No recurrence / death	HR (95% CI)	p-value
N		60	120		
Mean age in years (SD)		66.7 (9.8)	65.1 (8.5)	1.02 (0.98–1.06)	0.35
TNF inhibitor therapy after diagnosis (regardless of exposed prior)		8 (13.3%)	23 (19.2%)	0.75 (0.31–1.85)	0.54
Stage at diagnosis (row %)	**overall**				**0.03**
	stage 1	11 (18.3%)	28 (23.3%)	ref	
	stage 2	5 (8.3%)	26 (21.7%)	0.47(0.16–1.39)	0.17
	stage 3	5 (8.3%)	15 (12.5%)	1.33 (0.47–4.06)	0.62
	**stage 4 +**	**30 (50.0%)**	**32 (26.7%)**	**2.49 (1.06–5.89)**	** 0.04**
	unknown	9 (15.0%)	19 (15.8%)	0.92 (0.36–2.33)	0.85
Mean years from RA diagnosis to head and neck cancer diagnosis (SD)		11.4 (13.4)	9.4 (9.8)	1.10(0.99–1.24)*	0.09
Smoking, ever**		42 (70.0%)	63 (52.5%)	1.80 (0.97–3.33)	0.06
Alcohol, current**		29 (48.3%)	57 (47.5%)	0.94 (0.55–1.63)	0.83
Mean modified Romano score (SD)		5.5 (4.0)	4.9 (4.3)	1.03 (0.96–1.11)	0.38
**Radiation**		**44 (73.3%)**	**92 (76.7%)**	**0.35 (0.17–0.74)**	**0.01**
Chemotherapy		26 (43.3%)	37 (30.8%)	0.78 (0.37–1.62)	0.51
**Surgery**		**26 (43.3%)**	**67 (55.8%)**	**0.39 (0.20–0.76)**	**0.01**

* Natural log to correct for non-normality

** Measured at the time of HNC diagnosis

When subjects with evaluable stage were grouped by early (stage 1 and 2) and late (stage 3+) disease, those who received TNFi at any time (before or after HNC diagnosis) had a similar unadjusted rate of HNC recurrence or HNC-attributable death to those who only received nbDMARDs for early stage HNC (3/18, [17%] vs 14/52 [27%], p = 0.38), and significantly lower rate of unadjusted HNC recurrence or HNC-attributable death among those with late stage HNC (4/24, [17%] vs 27/60, [45%], p = 0.02). However, exposure to TNFi was not associated with either risk or protection from HNC recurrence or HNC-attributable death in our multivariate model as described above.

A sensitivity analysis restricted to biopsy-proven cancers, defined as those with documented histology (n = 178), showed similar results to our original analysis of 180 patients, with the same risk factors emerging as significant with similar hazard ratios. The sensitivity analysis showed that stage 4 cancer was a risk factor for recurrence or HNC-attributable death (HR 2.68 [CI 1.13–6.36]; p = 0.03); treatment with radiation or surgery was associated with a lower risk of recurrence or HNC-attributable death (HR 0.35 [CI 0.17–0.73]; p = 0.01 and HR 0.40 [CI 0.20–0.79]; p = 0.01 respectively).

## Discussion

In an observational cohort of RA patients with HNC derived from the US VA’s national administrative databases, we found that treatment with TNFi may not be associated with an increased risk of recurrence or death from head and neck cancer. Our data suggest that it may be safe to prescribe TNFi to patients with HNC and RA.

A few case reports have described HNC in patients on TNFi therapy. A patient with Crohn’s disease on infliximab therapy developed squamous cell carcinoma of the base of the tongue, which after a complete response to treatment with chemotherapy and radiation, recurred after resumption of infliximab for Crohn’s disease [8]. Another report described an epidermoid carcinoma of the buccal mucosa in a patient with RA detected after etanercept was initiated [9]. Another patient with RA on etanercept, previously treated with infliximab and adalumimab, developed two primary squamous cell carcinomas of the tongue 22 months apart, which responded to surgery and radiation, with discontinuation of etanercept [10]. We reported on a patient with ankylosing spondylitis who developed an invasive squamous cell carcinoma of the lower lip after two years of adalimumab therapy [11].

Although these case reports indicate that TNFi may influence the development and recurrence of HNC in patients with various inflammatory diseases, the effect of exposure to TNFi on the risk of recurrence or HNC-attributable death has not been previously examined in a rigorous manner. As expected, our study showed that stage at diagnosis and advanced stage disease adversely affected the risk of our composite outcome, and treatment with radiation and surgery was protective [12]. Smoking and alcohol use are important risk factors for HNC and traditionally have accounted for 80–90% of the risk for these cancers [13] Smoking and alcohol use, both current and past, was high in our cohort, but neither emerged as a risk factor for HNC recurrence or death. Advanced stage disease, that is, stage 3 and 4 disease, is a prognostic indicator of poor survival, as shown in a large series of four hundred and fifteen patients with HNC [14]. Surgery and radiation as treatment modalities may be surrogate markers of earlier, less advanced disease and were associated with decreased risk for HNC recurrence or death in our study.

Although the risk for overall malignancy with the use of TNFi in RA has been studied extensively, the risk for site or organ specific malignancies has not been examined as thoroughly, with the possible exception of skin cancer. As we and other investigators have shown, patients with RA on TNFi appear to be at an increased risk for non-melanoma skin cancer [2–4]. Such a risk may not extend to other solid tumors [3–5] including recurrence or HNC-attributable death, based on the results of this study. Two meta-analyses which examined this issue found that aside from non-melanoma skin cancer, the risk for other cancers in RA patients on TNFi was not increased [3,4].

Our study has several strengths. The ability to access and review the VA’s national patient electronic medical record databases permitted the construction of a validated cohort of RA patients with HNC who had or had not received a TNFi, and the evaluation of rare events such as HNC using stringent inclusion and exclusion criteria. Our study period of ten years allowed us to examine outcomes which may have long latency periods; in this case HNC recurrence or death. We examined a comprehensive list of potential risk factors including age, smoking and alcohol use, stage of cancer, treatment modalities, and a co-morbidity score as predictors of HNC recurrence or death. Our VA cohort was inherently enriched for cases of HNC because it consisted of predominantly male, Caucasian, older patients with RA who were smokers. These factors that usually limit the external validity of findings in a VA cohort became strengths in our study.

Our study has limitations as well. Given the observational nature of the study, there was a potential for channeling bias. If a patient was at higher risk of HNC recurrence (older age, non-Caucasian race, current smoker, recent diagnosis of HNC, advanced stage of HNC), a clinician may have been less likely to consider using TNFi in such a patient, making TNFi appear safe. The likelihood of such channeling bias is mitigated by the inclusion in our multivariate model of known risk factors for recurrence, but some bias toward the null may still be present. There is no generally accepted and validated standardized tool to measure RA severity in administrative data, and RA severity may impact HNC recurrence or death; we could not examine this variable as a risk factor given the retrospective nature of our study. We were unable to examine the role of human papilloma virus infection, an important risk factor for HNC [15] given the lack of information on this in our retrospective database.

Finally, our results may have been biased by unmeasured confounders such as disease duration, dose of and compliance with DMARDs, lack of quantification of tobacco and alcohol exposure and inability to include them as time-varying co-variates, the relatively small sample size, and missing data on tumor histology and staging in some cases; all unavoidable pitfalls because of the observational design of the study. Some veterans may have been treated outside the VA system leading to information or classification bias. As this was an observational study, and as some veterans use both VA and non-VA services, the duration of follow-up was variable. Therefore it is likely that some events were missed because these occurred outside of VA care. Furthermore, more recent prescriptions for TNFi or nbDMARDs would have had less follow-up time to detect HNC recurrence or HNC-attributable death. These factors may have not allowed an accurate estimation of the risk conferred by TNFi on recurrence or HNC-related death in this cohort. However, the large size of the VA administrative databases allowed us to assemble the largest cohort with RA and HNC to our knowledge to date. While the cohort was relatively small, it is unlikely that such a cohort could be prospectively collected due to the uncommon co-occurrence of these diseases. Ideally, results from this observational study should be validated in larger, prospective, well-defined cohorts where some of these confounders can be eliminated.

## Conclusion

The results of this study suggest that TNFi use may be safe, and not associated with an increased risk of recurrence or HNC-attributable death in patients with RA. Because of concerns about the potential risk of malignancy recurrence with TNFi, many clinicians tend to avoid using these drugs in RA patients with a history of malignancy. Based on our results, these agents may be safe in patients with RA and HNC with close monitoring, especially as the time interval between HNC treatment and non-recurrence increases. Given the near ubiquitous use of TNFi in RA, there is a compelling need for further safety studies of TNFi in patients with malignancy.
